# Discovery of Chlorophyll *d*: Isolation and Characterization of a Far-Red Cyanobacterium from the Original Site of Manning and Strain (1943) at Moss Beach, California

**DOI:** 10.3390/microorganisms10040819

**Published:** 2022-04-14

**Authors:** Nancy Y. Kiang, Wesley D. Swingley, Dikshyant Gautam, Jared T. Broddrick, Daniel J. Repeta, John F. Stolz, Robert E. Blankenship, Benjamin M. Wolf, Angela M. Detweiler, Kathy Ann Miller, Jacob J. Schladweiler, Ron Lindeman, Mary N. Parenteau

**Affiliations:** 1 NASA Goddard Institute for Space Studies, New York, NY 10025, USA; 2 Virtual Planetary Laboratory, Nexus for Exoplanet System Science, NASA Astrobiology Program, USA; 3 Department of Biological Sciences, Northern Illinois University, DeKalb, IL 60115, USA; gautam.dikshyant@gmail.com (D.G.); jjschlad1408@gmail.com (J.J.S.); 4 NASA Ames Research Center, Moffett Field, CA 94035, USA; jared.t.broddrick@nasa.gov (J.T.B.); angeladetweiler@gmail.com (A.M.D.); 5 Woods Hole Oceanographic Institution, Woods Hole, MA 02543, USA; drepeta@whoi.edu; 6 Department of Biological Sciences, Duquesne University, Pittsburgh, PA 15282, USA; stolz@duq.edu; 7 Center for Environmental Research and Education, Duquesne University, Pittsburgh, PA 15282, USA; 8 Departments of Biology and Chemistry, Washington University in St. Louis, St. Louis, MO 63130, USA; reblankenship@gmail.com (R.E.B.); wolfbenjamin25@gmail.com (B.M.W.); 9 Bay Area Environmental Research Institute, Moffett Field, CA 94035, USA; 10 University Herbarium, University of California, Berkeley, CA 94720, USA; kathyannmiller@berkeley.edu; 11 Citizen Scientist, Alameda, CA 94501, USA; ron.lindeman123@gmail.com

**Keywords:** chlorophyll *d*, *Acaryochloris*, Moss Beach, cyanobacteria, far-red photosynthesis, photosynthetic pigments, absorbance spectra, genome sequence

## Abstract

We have isolated a chlorophyll-*d*-containing cyanobacterium from the intertidal field site at Moss Beach, on the coast of Central California, USA, where Manning and Strain (1943) originally discovered this far-red chlorophyll. Here, we present the cyanobacterium’s environmental description, culturing procedure, pigment composition, ultrastructure, and full genome sequence. Among cultures of far-red cyanobacteria obtained from red algae from the same site, this strain was an epiphyte on a brown macroalgae. Its Q_y_ *in vivo* absorbance peak is centered at 704–705 nm, the shortest wavelength observed thus far among the various known *Acaryochloris* strains. Its Chl *a*/Chl *d* ratio was 0.01, with Chl *d* accounting for 99% of the total Chl *d* and Chl *a* mass. TEM imagery indicates the absence of phycobilisomes, corroborated by both pigment spectra and genome analysis. The Moss Beach strain codes for only a single set of genes for producing allophycocyanin. Genomic sequencing yielded a 7.25 Mbp circular chromosome and 10 circular plasmids ranging from 16 kbp to 394 kbp. We have determined that this strain shares high similarity with strain S15, an epiphyte of red algae, while its distinct gene complement and ecological niche suggest that this strain could be the closest known relative to the original Chl *d* source of Manning and Strain (1943). The Moss Beach strain is designated *Acaryochloris* sp. (*marina*) strain Moss Beach.

## 1. Introduction

Chlorophyll *d* (Chl *d*) is a far-red-absorbing chlorophyll, which Manning and Strain [1] first discovered off the coast of Central California, and assumed to be a pigment of the red macroalgae growing in the intertidal zone. It was not until more than 50 years later that Miyashita, et al. [2] found Chl *d* to be not an accessory pigment of algae, but a primary photopigment of a cyanobacterium, which they named *Acaryochloris marina* (type-strain MBIC11017). They discovered that Chl *d* replaces nearly all chlorophyll *a* (Chl *a*) in these organisms, enabling them to perform oxygenic photosynthesis at long wavelengths in the far-red/near-infrared [3]. This discovery overturned long-held wisdom about the primacy of chlorophyll *a* and the photon energy limits for oxygenic photosynthesis.

Li and Chen [4] and Allakhverdiev, et al. [5] provided detailed reviews of the molecular structures of the different chlorophylls, and of research to characterize the position and function of Chl *d* in Photosystems I and II (PSI and PSII). Whereas Chl *a* possesses a vinyl group at the C3 position of the chlorin macrocycle, with its Soret and Q_y_ bands peaking in absorbance at 435 and 666 nm, respectively, in methanol, Chl *d* instead possesses a formyl group, such that its Soret band is broadened with two peaks at 401 and 460 nm, and the Q_y_ peak is at 697–698 nm. PSI in *A. marina* turns out to have a Chl *d* homodimer for the special pair [6], while in Photosystem II (PSII) it remains to be confirmed whether the special pair is a Chl *a*/Chl *d* heterodimer [7] or a Chl *d*/Chl *d* homodimer [8]. Mielke, et al. [9] resolved questions as to whether *A. marina* could have inefficiencies in the use of long-wavelength photons due to recombinations, showing that the photon-energy-storage efficiency in *A. marina* is comparable to or higher than that of Chl *a* organisms. They found *A. marina* MBIC 11017 had 40 ± 1% thermal storage efficiency compared to that measured in Chl *a* organisms (ranging from 34.1 ± 1% in *Synechococcus leopoliensis* to 43 ± 2% in an alga, *Chlorella vulgaris*, Cha and Mauzerall [10]). It has been estimated through photoacoustics that the PSII trap wavelength is 723 ± 3 nm [11], and through laser flash absorption that the PSI trap is at 740 nm [12]; such that they are 40–46 nm and 40 nm to the red of their Chl *a* counterparts, respectively.

The discovery of *Acaryochloris* has motivated a hunt for more far-red oxygenic phototrophs and led to a proliferation of discoveries, including: additional strains of *Acaryochloris* [13,14,15,16]; numerous cyanobacteria that utilize Chl *d* as well as chlorophyll *f* (Chl *f*) [17] at yet longer wavelengths for uphill energy transfer to Chl *a* in Far-Red Light Photoacclimation (FaRLiP) [18,19,20]; and even a far-red Chl *a* as a photopigment in an alga [21]. All of these organisms live in niche environments depleted in visible light and relatively enriched in the far-red/near-infrared, receiving light filtered by Chl-*a*-containing organisms. *A. marina* str. MBIC11017 [2] was found off the coast of the Palau Islands under an ascidian that contains a Chl-*a*-utilizing cyanobacterial symbiont [22]. Murakami, et al. [15] found *A. marina* sp. strain Awaji to be an epiphyte of red algae at Awaji Island, Japan; this solved the original puzzle about whether str. MBIC11017 was a symbiont or epiphyte of its ascidian [22]. Larkum, et al. [13] later found another *Acaryochloris* epiphyte of red algae in a mangrove at Salt Pan Creek, Georges River NSW, Australia, *Acaryochloris* sp. MPGRS1. Miller, et al. [16] found *A. marina* str. CCMEE 5410 from a microbial mat rock biofilm in the Salton Sea, California, USA. Mohr, et al. [14] obtained *Acaryochloris* sp. HICR111A from scrapings off of a dead coral skeleton overgrown with other algae and cyanobacteria at Heron Island, Great Barrier Reef, Australia. Mehda, et al. [23] found cf. *Acaryochloris* in Sahara Desert biocrusts through phylogenetic analysis. Most recently, Ulrich, et al. [24] isolated strain S15 from a red alga off the coast of Northern California, USA. Numerous additional 16S rRNA entries of unpublished *Acaryochloris* discoveries can now be found in the databases of the Ribosomal Database Project (RDP) (https://rdp.cme.msu.edu) and the National Center for Biotechnology Information (NCBI) (https://www.ncbi.nlm.nih.gov).

Despite these recent discoveries, no one, to our knowledge, has returned to Manning and Strain’s site of the original discovery of Chl *d*, until now. Thus far, full genome sequences have only been obtained for *A. marina* MBIC11017 [25], *Acaryochloris* sp. CCMEE 5410 [16], and *A. marina* S15 [24]. Here, we present a strain of *Acaryochloris* from Moss Beach, CA, with a description of its isolation and culture methods, its pigment composition, ultrastructure, and a full genome sequence. We compare these results to other strains and species in the literature, and analyze the Moss Beach strain’s genomic characteristics and its place in the *Acaryochloris* phylogenetic tree.

## 2. Materials and Methods

**Figure 1:**
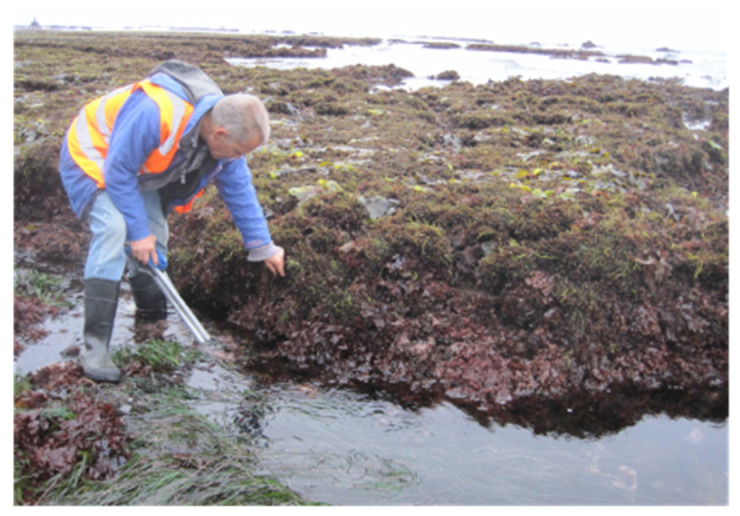
Rocky intertidal zone field site at low tide on 2 August 2015, J.V. Fitzgerald Marine Reserve, Montara State Marine Reserve. Moss Beach, CA, USA. Shows depth layering of green and red macroalgae. (Pictured: Ron Lindeman).

The original site of the Manning and Strain [1] discovery of chlorophyll *d* is a rocky intertidal zone at Montara State Marine Reserve, Moss Beach, CA, USA (122°30′55.7″ W 37°31′15.3″ N). Water temperatures range from 9–15 °C seasonally, and algal growth peaks in summer, with a particular abundance of red macroalgae (Figure 1).

### 2.1. Establishment of Cultures

On 2 August 2015, a variety of green, red, and brown macroalgae was collected at low tide at the J.V. Fitzgerald Marine Reserve protected area of Montara State Marine Reserve, Moss Beach, California, during the peak growing season for the red algae (Figure 1). Clippings of each sample were used to inoculate enrichment cultures in iron-enriched marine BG-11 growth medium, “FeMBG-11” [26] consisting of 10.4 mg/L EDTA and iron(III) monosodium salt (Fe-EDTA) added to 3.5% Instant Ocean artificial sea salts (Aquarium Systems, Mentor, OH, USA), and with trace minerals, ferric ammonium citrate, bicarbonate, potassium phosphate, as published previously [27]. However, instead of N-[Tris(hydroxymethyl)methyl]-2-aminoethanesulfonic acid (TES) buffer, we used Trizma HCL buffer adjusted to pH 8.2 with NaOH pellets prior to autoclaving; after autoclaving, the pH of the medium was typically above 8.7 to 9.0. In addition, enrichment cultures were augmented with “f/2” vitamins modified from the “f-1” recipe of Guillard and Ryther [28], consisting of 0.25 µg/L cyanocobalamin vitamin B12, 0.25 µg/L biotin vitamin H, and 0.2 mg/L Thiamin vitamin B-1.

**Figure 2:**
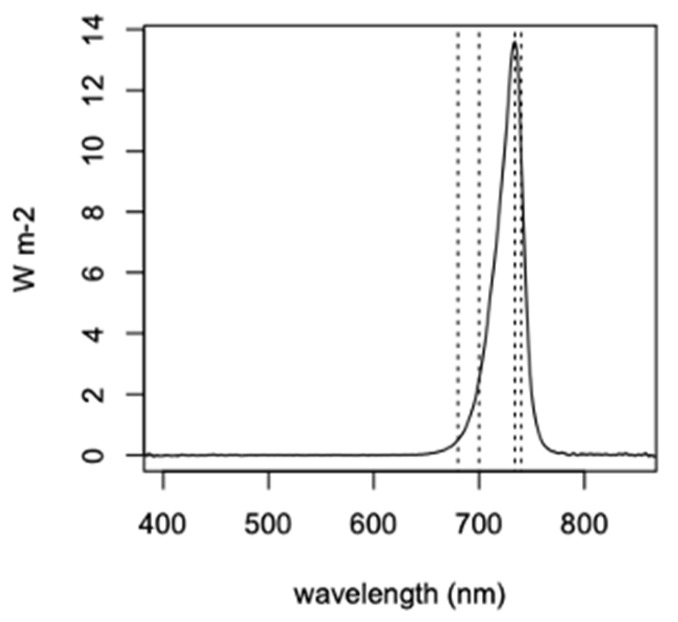
~730 nm LED light bar irradiance spectrum to a central position in the *Acaryochloris* growth chamber. Dashed lines mark wavelengths at 680 (P680), 700 (P700), 733.7 (LED peak), and 740 nm (P740). Over 400–700 nm, the integrated flux is 1.74 µmol m^−2^ s^−1^. Over 400–740 nm, the integrated flux is 21.87 µmol m^−2^ s^−1^ and 3.63 W m^−2^.

The cultures were incubated at room temperature under a ~730 nm light-emitting diode (LED) light (LED Light Bar 730 nm Far Red, BML Horticulture, Austin, TX, USA), with irradiance over 400–740 nm ranging from 12–22 µmol m^−2^ s^−1^. with a 12 h/12 h light/dark cycle. The spectrum for the LED, as measured with a JAZ-A-Irrad absolute irradiance spectroradiometer (Ocean Optics, Inc., Dunedin, FL, USA) at an irradiance location central to the growth chamber, is shown in Figure 2. The exact peak of the LED light bar is at 733.7 nm, and dashed lines demark wavelengths at 680, 700, 733.7, and 740 nm. This shows there is non-zero photon flux below 680 and 700 nm, such that photosynthesis with Chl *a*-based P680 and P700 could still receive a small amount of photosynthetically active radiation (PAR, 400–700 nm), but otherwise 93% of the 400–740 nm irradiance in photon flux is in the far red, beyond the range of P700. The intensity of the light bar was adjusted such that irradiance over 400–740 nm incident to the samples ranged between 11–22 µmol m^−2^ s^−1^.

We unsuccessfully attempted to establish axenic cultures from the enrichments using serial dilutions to extinction using inoculum that was sonicated to break up clumps of cells. However, we were successful in establishing unialgal cultures by selecting single-pigmented colonies on Petri plates containing FeMBG-11 medium hardened with 0.4% agarose. To generate more biomass for subsequent pigment and physiological characterizations, pigmented colonies from a plate were used to inoculate Erlenmeyer flasks ranging from 25 mL to 1 L, in the latter maintaining a medium depth of no more than 1.5 inches to allow for greater surface area of the medium for CO_2_ exchange and diffusion to the bottom of the flask. Subsequent to genome sequencing, further maintenance of the cultures included antibiotic treatment to remove contaminating chemotrophic organisms. A plate colony was treated with an antibiotic mix of 25 µg/mL tetracycline, 25 µg/mL kanamycin, 100 µg/mL ampicillin, incubated in the dark for 24 h, and then the cells were transferred to regular FeMBG-11 growth medium. Biomass was preserved in −80 °C in the spent growth medium, and the maintained cultures descend from this antibiotic treatment.

### 2.2. In Vivo Spectral Absorbance

*In vivo* spectral absorbance was obtained as follows: 200 µL, 500 µL, and 800 µL aliquots of cultures were diluted into 10 mL of 10 mM Tris 50 mL NaCl pH 8.0 and gently vacuum-filtered onto 2.4 cm diameter GF/C filters. The filters were placed on top of a 96-well microtiter plate and their absorbance spectrum was measured on a Molecular Devices SpectraMax M5 spectrometer, at a spectral resolution of 1 nm over 350–900 nm. As a blank, 10 mL of buffer was filtered on GF/C filters and analyzed in the same manner, and was used to correct the baseline of the *in vivo* spectral absorbance. For each filter, 2 spots were analyzed as technical replicates and averaged together. The absorbance readings of the 200 µL and 500 µL were corrected to a total volume of 800 µL, the optical density (OD) values were averaged, and the standard deviation was determined.

### 2.3. Pigment Characterization

Pigment analysis was performed on samples of whole, intact cells of the far-red cyanobacteria cultures enriched from the fronds of the brown macroalgae *Stephanocystis osmundacea* (sample A-19), which were centrifuged into pellets in growth medium, and stored at −80 °C in growth medium prior to thawing for pigment extraction.

**Pigment Extraction.** The pigments in the cell pellets were recovered by sequential sonic extraction alternating between 0.5 mL of 100% methanol and 0.5 mL of 100% acetone (10 min each) until the final extract was not visibly colored. Cells were resuspended before each sonication by vortex mixing. The extracts were combined and reduced to a small volume (~1 mL) by rotary vacuum evaporation at room temperature.

**High-performance liquid chromatography (HPLC).** Culture extracts were analyzed for pigments on an Agilent 1200 HPLC system fitted with a diode-array detector. Pigments were separated on an Ascentis C-18 column (150 × 2.1 mm; 3 µm) eluted at 300 µL min^−1^ using a linear gradient from 100% solvent A (80%/20% *v*/*v* methanol/aqueous 50 mM ammonium acetate) to 100% solvent B (80/20 *v*/*v* methanol/acetone) over 25 min followed by isocratic elution at 100% B for an additional 30 min. Pigments were detected between 400–750 nm.

**Biomass.** The extracted biomass used for pigment analyses was then air dried and weighed to obtain dry biomass. The dry biomass was then used to calculate the moles Chl *d* per grams biomass. We note that these estimated dry weights represent a minimum in biomass dry weight, and therefore a slight overestimate of pigment per gram of cell dry biomass, as they do not account for the organic matter that was extracted from the sample by the methanol and acetone. Acetone and methanol will extract lipids, which are ~10–20% of the cell dry weight, as a rule of thumb [29]. Therefore, the Chl/biomass presented here is a maximum and may be an overestimate by 10–20% since it derives from an underestimate of total biomass. However, this will not affect the estimated Chl *d*/Chl *a* ratio.

**Calibration for Chl *d* extinction coefficient.** Chl *d* and Chl *a* were purified by HPLC using the same column and flow rate detailed above, but with isocratic elution with 100% solvent B. The Chl *d* fraction (3.8–4.2 min) was collected and dried by rotary vacuum evaporation at room temperature. Spectral absorbance of the purified Chl *d* in 100% methanol was measured on an Evolution 300 spectrophotometer over 350–800 nm with a spectral resolution of 1 nm. The mass extinction coefficient $\alpha_{\lambda }$ = 71.11 (L g^−1^ cm^−1^) for Chl *d* obtained by Li, et al. [30] and the 697 nm absorbance were used to obtain the mass concentration C (g L^−1^) of Chl *d* in the solution. This solution was used to calibrate the HPLC detector response factor. For Chl *a*, the mass extinction coefficient of 88.15 (L g^−1^ cm^−1^) [31] and prior calibration of the HPLC were used to obtain the mass concentration of Chl *a*.

### 2.4. Transmission Electron Microscopy (TEM)

Cells were prepared for TEM using a modification of the method in Switzer Blum, et al. [32]. The cells were initially fixed with glutaraldehyde (2.5% final concentration) in their culture medium for 2 h. They were then rinsed 3 times in Phosphate Buffered Saline (PBS) with 6% sucrose, then post fixed for 1 h with 2% osmium tetroxide (0.5 M sodium acetate). Following a triple rinse with 0.5 M sodium acetate buffer, the cells were incubated overnight with 0.5% uranyl acetate. The cells were then dehydrated in an ethanol series (50%, 70%, 90%, 95%, and 100%), followed by propylene oxide treatment (first straight, then 1:1 with Spurr’s low-viscosity embedding medium) and embedded in Spurr’s. Ultrathin sections were observed on a JEOL 1210 TEM (JEOL, Peabody, MA, USA) at 80 kV equipped with an ORCA HR digital camera (Hamamatsu, Bridgewater, NJ, USA).

### 2.5. DNA Extraction

Approximately 10 mL of cells in mid-exponential growth phase were pelleted by centrifugation. Cells were washed with 1 mL of 50 mM Tris HCl, pH 7.5, and pelleted a second time by centrifugation. The resulting pellet was reconstituted in 370 µL of 50 mM Tris HCl, 100 mM NaCl, 20 mM EDTA, 300 µg RNase A, and 400 µg lysozyme, and incubated in a water bath at 58 °C for 1 h. To this mixture, 200 µg proteinase K and 20 µL of 20% SDS (final concentration 1%) were added and incubated an additional 2 h at 58 °C, inverting several times during the incubation. The mixture was then cooled for 5 min in an ice bath before pelleting residual cell debris by centrifugation at 10,000× *g* for 10 min. To the supernatant, a solution of 150 mM Tris HCl pH 7.5, 40 mM EDTA, 4% cetyl trimethyl ammonium bromide (CTAB) and 2.4 M NaCl was added in a 1:1 ratio and incubated at 58 °C for 15 min. The mixture was cooled on ice for 5 min, combined with 600 µL of chloroform and mixed by inversion for 3 min. Phase separation was achieved by centrifugation at 10,000× *g* for 10 min and the upper aqueous phase was transferred to a new 1.5 mL microcentrifuge tube.

An additional RNA removal step was accomplished by adding 200 µg of RNase A and incubating at room temperature for 15 min before performing a second chloroform addition (500 µL). After mixing and centrifugation, the aqueous phase was transferred to a new 1.5 mL microcentrifuge tube. DNA was precipitated by adding 2 volumes of a solution containing 100 mM Tris HCl pH 7.5, 20 mM EDTA and 2% CTAB. DNA was pelleted by centrifugation and washed with 1 mL 70% ethanol (EtOH). This genomic DNA (gDNA) pellet was washed again with 1 mL 70% EtOH for 30 min on a rotary mixer. The gDNA pellet was then washed a final time with 1 mL 70% EtOH before being reconstituted in 15 µL 10 mM Tris HCl, 50 mM NaCl pH 7.5 in nuclease-free water. DNA quality was assessed by fluorometric quantification (Qubit, Invitrogen, Waltham, MA, USA) and UV-Vis microvolume spectrophotometry (Nanodrop, Thermo Fischer Scientific, Waltham, MA, USA). All gDNA manipulation was performed using wide-bore pipet tips to prevent gDNA shearing.

### 2.6. DNA Sequencing

Native genomic DNA (gDNA) was sequenced on a MinION R9.4 flowcell (Oxford Nanopore Technologies (ONT), Oxford, UK). The sequencing library was prepared using the ONT Rapid Barcoding Sequencing kit (SQK-RBK004) according to the manufacturer’s protocol, with the following modifications: two separate 0.2 mL PCR tubes, 1 and 0.5 μg gDNA, were diluted to 9 μL in ONT EB (10 mM Tris, 50 mM NaCl, pH 7.5). The barcoded fragmentation mix was added in a ratio of 3:1 and 1:1 (μg gDNA:μL fragmentation mix) to the 1 μg and 0.5 μg samples, respectively. Half the library (~0.75 μg) was loaded onto the MinION flowcell. ONT EB was used to bring the total library volume to 75 μL prior to loading. Sequencing was performed for 5 h on a flowcell with approximately 1700 active pores. Sequencing Fast5 files were base-called using the ONT Guppy base-caller (v3.2.2) with GPU acceleration on a laptop with an Intel i7-6550U processor and 8 GB RAM connected to external GPU housing with an Nvidia GTX1070 (1920 CUDA cores, 8 GB VRAM) via a Thunderbolt 3 connection. Quality filtering was enabled with default settings using the high-accuracy base-calling algorithm.

An Illumina library was made using the Nextera kit (Illumina, San Diego, CA, USA) following the protocol recommended by the manufacturer and then pair-end sequenced using shotgun sequencing on the HiSeq 2500 platform at the University of Illinois at Chicago (Chicago, IL, USA) Genome Research Core DNA services facility.

### 2.7. Genome Sequence Assembly and Analysis

Nanopore reads were assembled using Flye v2.6 [33] with the arguments “–plasmids –meta–asm-coverage 50–min-overlap 2500”. As the original culture was unialgal but not axenic, the resulting contigs were filtered to identify those likely to originate from members of the genus Acaryochloris by comparing predicted proteins identified using PRODIGAL v2.6.3 [34] to the NCBI nr database using BLASTP in the Basic Local Alignment Search Tool (BLAST) 2.5.0+ [35]. The top five hits for each protein, based on E-value, were used to filter out any contigs that coded for a majority of proteins (>50%) with no matches to Acaryochloris strains in their top five hits. Filtered contigs were cleaned twice with Pilon v1.23 [36] using paired Illumina reads to create a hybrid assembly. Contigs were manually verified to be circular by identifying overlapping regions at the start and end of linear FASTA sequences using BLASTN. Redundant sequence information present at the beginning and end of any contigs was removed. Coverage from Nanopore reads was extracted from the output file of the Flye assembler, while the coverage from Illumina reads were extracted using bwa 0.7.5a-r405 [37] followed by Samtools v1.9 [38] to map reads back to assembled contigs.

Final published annotation was generated through NCBI using their NCBI Prokaryotic Genome Annotation Pipeline (PGAP). The resulting proteins obtained from PGAP were used to calculate the amino acid identity (AAI) shared with other publicly available species of *Acaryochloris* (accession numbers in Table S1; access date 1 December 2021) using CompareM v0.1.1 (https://github.com/dparks1134/CompareM). Insertion elements were calculated using isescan 1.7.2. [39] using its default settings. CRISPR elements were identified using CRISPRCASTyper [40].

Visualization of the *Acaryochoris* sp. Moss Beach genome and its plasmids versus closely related strains was generated using the CGView_comparison tool [41]. The scripts cgview_comparison_tool.pl and redraw_maps.sh were used, and the configuration file project_settings.conf was modified to generate the comparative analysis. The gene content and synteny of *Acaryochloris* sp. Moss Beach, *A. marina* MBIC11017, and *A. marina* S15 [24,26] were visualized using Artemis Comparison Tool (ACT) v18.1.0 [42], using BLASTN results to display similarity, insertions, and rearrangements across the entire genomes. Finally, reciprocal BLAST hits were generated between strains Moss Beach and S15 using BLASTP and a custom python script to calculate and display orthologous proteins between these closely related strains.

## 3. Results

### 3.1. Cultures

The species of red and brown macroalgae that were collected on the field trip of 2 August 2015, are listed in Table 1. We note that some of these species can appear very green, but are classified as red or brown. *Neogastroclonium* and *Chondracanthus* are very dark green, and *Mazzaella flaccida* bright green, but they are red algae. *Desmarestia* is a brown alga that turns green when out of water, because H_2_SO_4_ in its cells is released and destroys its brown pigments. The table marks which samples contained Chl *d* by directly solvent-extracting from algal clippings (analyzed via HPLC) (“p”), and which samples yielded successful cyanobacterial cultures (“c”). The enrichment in far-red LED light and FeMBG-11 growth medium produced growth of far-red cyanobacteria for 16 of 26 inoculants from the August samples. While solvent extracts of clippings did not necessarily yield Chl *d* for some samples (likely due to low concentrations), the same algal sample sometimes yielded far-red enrichment cultures; likewise, algal clippings that had the presence of Chl *d* did not always yield viable cyanobacterial cultures.

**Figure 3:**
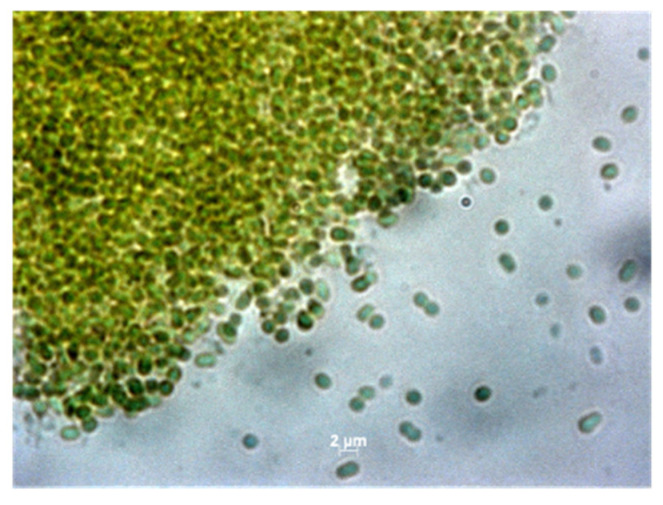
Phase contrast microscope images at 100X magnification showing cells of the *Acaryochloris* sp. culture from Moss Beach, CA, USA.

The cultures exhibited several different modes of growth: benthic biofilm, planktonic suspensions, and clumped cells settled on the bottom of the flask. Due to slow growth and the difficulty of establishing pure cultures of each enrichment, we focused on characterizing one culture originating from A-19. We continued to maintain only cultures from a few lineages, as highlighted in Table 1. Cells from *A-19* as well as from cultures from other algal samples were unicellular, ovoid, 2–3 µm long, and 1.5–2 µm in diameter (Figure 3).

**Figure 4:**
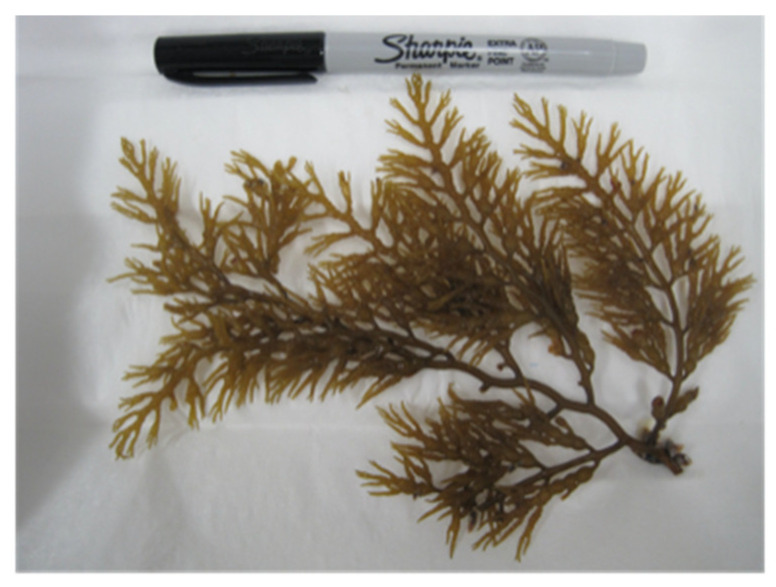
Brown macroalgae *Stephanocystis osmundacea*, sample A-19 from the 2 August 2019, field trip and source of inoculum for the *Acaryochloris* culture analyzed in this study.

TEM images, spectral characterizations, and genome sequencing were conducted on the lineage originating from the inoculation from a clipping of *Stephanocystis osmundacea* (A-19), a brown macroalga with the popular name “chainbladder kelp” (Figure 4).

### 3.2. Pigment Characterization

**Figure 5:**
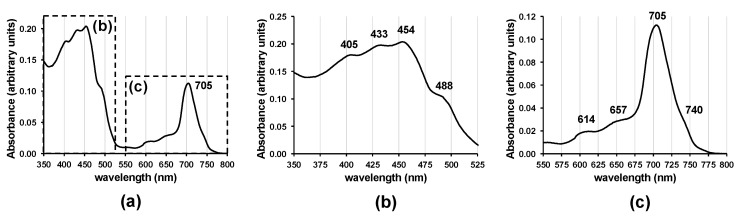
*In vivo* absorbance spectrum of whole cells of *Acaryochloris marina* strain Moss Beach on filter paper at a spectral resolution of 1 nm, (**a**) over 350–800 nm; (**b**) closeup over 350–525 nm; (**c**) closeup over 550–800 nm. From a culture originating from inoculation from a clipping of *Stephanocystis osmundacea*.

***In vivo* spectral absorbance.***In vivo* spectral absorbance of our *Acaryochloris* culture cell contents (Figure 5) exhibited a Q_y_ band peak absorbance at 705 nm. The two Soret band peaks at 405 and 454 nm may be of Chl *d* *in vivo*. There appears to be a slight shoulder at 740 nm.

**Figure 6:**
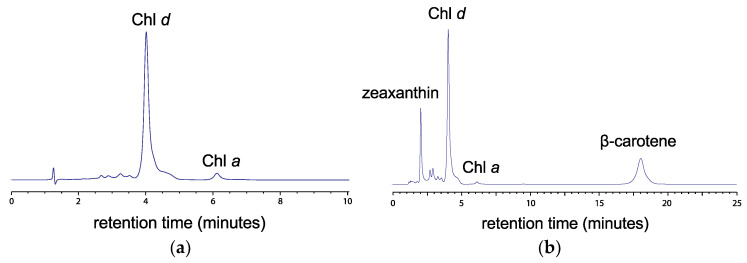
HPLC chromatograms for *Acaryochloris marina* strain Moss Beach (**a**) at 660 nm and (**b**) at 440 nm.

**Pigment separation and quantification.** The HPLC chromatograms are plotted in Figure 6 on the basis of Chl *a* Q_y_ absorption (at 660 nm) and Chl *a* Soret band absorption (at 440 nm). The chromatography allowed good separation of zeaxanthin (2.0 min), Chl *d* (~4.1 min), Chl *a* (~6.3 min), and ß-carotene (18.8 min). It is possible that the peak that we identified as ß-carotene could be or could include α-carotene, which has a very similar retention time and visible spectrum, but we did not have separate standards for each at the time. Since α-carotene is rarely seen in seawater samples, it is most likely the peak at 18.8 min is ß-carotene. A shoulder shortly after the peak for Chl *d* had a retention time less than a minute after, which could be consistent with the presence Chl *d’*, which is known to occur in PS I in MBIC11017 [5,43,44,45,46], or a degradation product. However, the absorbance spectrum of the shoulder shows no difference from Chl *d*.

**Figure 7:**
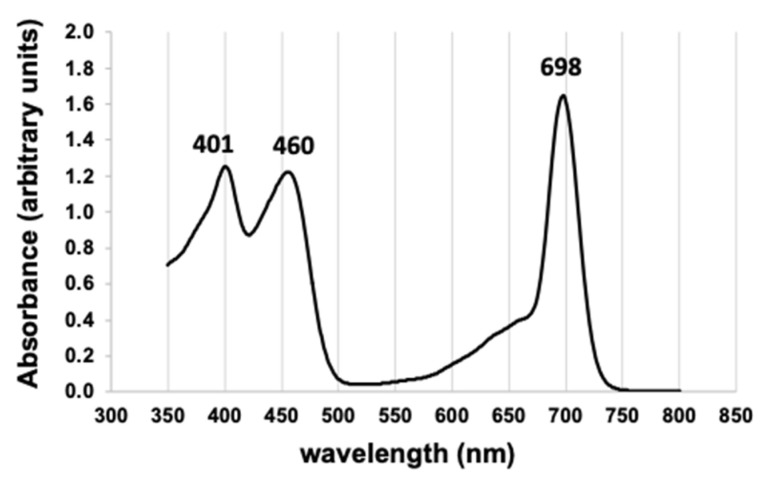
Absorbance spectrum of purified Chl *d* in 100% methanol, with wavelengths of local peak absorbance indicated.

**Chl *a*/Chl *d* ratio.** The spectral absorbance of the purified Chl *d* in 100% methanol (Figure 7) matched those of Li, et al. [30]. The Chl *a*/Chl *d* ratio was 0.01, or Chl *d* comprised 99% of the total mass of Chl *a* and Chl *d*. The mass of Chl *d* per cell biomass obtained was 28.9–30.4 µg-Ch *d*/g biomass (2 samples), or 32.2–33.9 mol-Chl *d*/g biomass based on an average Chl *d* molecular weight of 895.5 [30], and these actual values may be 10–20% lower given the underestimate of total biomass from the extraction procedure. However, the estimated Chl *d*/Chl *a* ratio was not affected by these systematic biases in the total biomass estimate.

### 3.3. TEM Images

**Figure 8:**
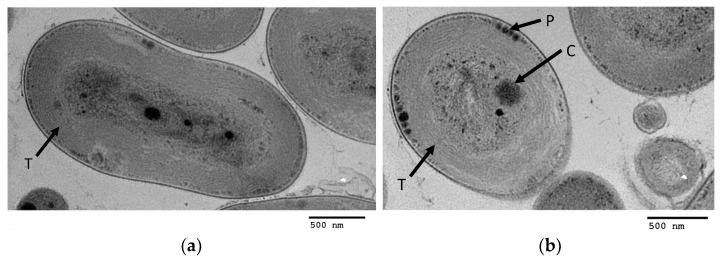
Oblong (**a**) and transverse (**b**) cross-sectional images of cells by transmission electron microscopy (TEM) showing the parietally arranged thylakoid membrane stacks (T) and polyphosphate granules (P) lined up along the cytoplasmic membrane of the cell wall, and carboxysome (C) in the center of the cell.

TEM images (Figure 8) show the Moss Beach cells to be rod-shaped, ~1.3 µm in diameter, and ~2.6 nm in length. The cell sizes are similar to the other strains described [2,16]. Parietally arranged thylakoid membranes line the cell-wall interior [47]. Other notable features are the presence of carboxysomes (Figure 8b) in the cytoplasm and polyphosphate globules between the cytoplasmic membrane and the thylakoids.

### 3.4. Genome Statistics

The complete genome assembly of *Acaryochloris* sp. strain Moss Beach yielded a circular chromosome of 7.25 Mbp and 10 circular plasmids ranging from 16 kbp to 394 kbp. This is consistent with the genome of type-strain *Acaryochloris marina* MBIC11017, at 8.36 Mbp and 9 plasmids, as summarized in Table 2 for genome features and Table S2 for guanine-cytosine (GC) content. The average coverage depth for nanopore sequence data was 174X and Illumina polishing coverage was estimated at 7X coverage. The GC average for the entire genome (the chromosome and all 10 plasmids) was 45%, with an average coding density of 78%. The genome codes for 5843 open reading frames, smaller than the 8528 for *A. marina* (Table S2). The genome size and statistics are consistent with other published strains [24,25,48,49], though the 10 plasmids in strain Moss Beach are the highest of any strain yet sequenced. CRISPR-Cas analysis on the Moss Beach strain showed that it lacks the *cas1* and *cas2* genes, consistent with previous studies in *Acaryochloris* [50], which showed that they lacked a *cas* operon and multiple spacer units.

### 3.5. Relationship of Strain Moss Beach with Other Acaryochloris Strains

*Acaryochloris* sp Moss Beach shares the highest average amino acid identity (AAI) with strain S15 [24], at ~97%, which is higher than the shared AAI with strains CCMEE 5410 and MBIC11017 at 89.1% and 88.9%, respectively (Table 3). The only sequenced *Acaryochloris* strain that does not produce the genus’ signature Chl *d*, *Acaryochloris thomasi* RCC1774 [49], shares just ~65% AAI with the Moss Beach strain, reinforcing its position as a deeply branching member of the genus, or perhaps suggesting that RCC1774 is not actually a true member of this genus. Finally, a set of draft (i.e., incomplete) metagenome-assembled genomes, SU_5_25, CRU_2_0, and RU_4_1 [51], share between 75–76% AAI with strain Moss Beach (Table 3).

**Figure 9:**
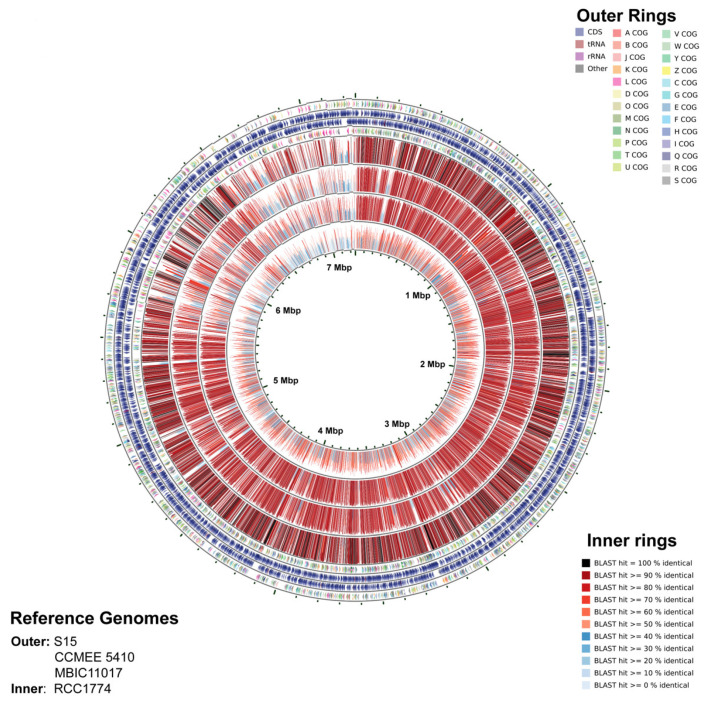
CGview Comparison Tool map representing the annotation of *Acaryochloris marina* strain Moss Beach (outer narrow rings) and BLAST comparison to *A. marina* S15 (outer wide band), *Acaryochloris* sp. CCMEE 5410 (next inward), *A. marina* MBIC11017 (next inward), and *Acaryochloris thomasi* RCC1774 (innermost wide band). In order from the outermost ring, each ring represents: Ring 1 and 4, COG functional categories for forward strand and reverse strand CDS; Ring 2 and 3, forwards and reverse strand sequence features; Rings 5–8, BLAST results (highest identity) compared to *Acaryochloris* genomes referenced above.

Coding sequences (CDS) in strain Moss Beach are generally consistent with other Chl *d*-producing strains (Figure 9), with greater than 90% of CDS on the main chromosome shared with other strains. However, this continuity was much lower with RCC1774 (Figure 9**,** inner ring). While much of the Moss Beach chromosome shares the highest similarity to strain S15 (Figure S1), there are regions where the other two Chl *d* strains have higher similarity; for example, eight o’clock in Figure 9. This disruption in the close relationship between Moss Beach and S15 is more apparent in the plasmids, roughly 9 to 12 o’clock, whereas the Moss Beach strain shares either a lower similarity to S15 than other strains, or no similarity to any sequenced *Acaryochloris*. Further comparison of individual plasmids to S15 and MBIC11017 is shown in Figure S2.

**Figure 10:**
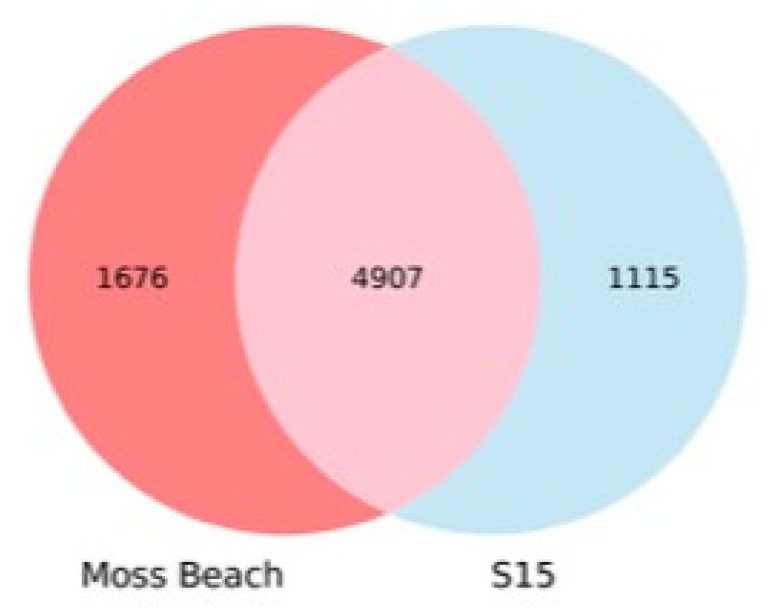
Reciprocal best BLAST hits for orthologous protein in *A. marina* strains Moss Beach and S15.

**Figure 11:**
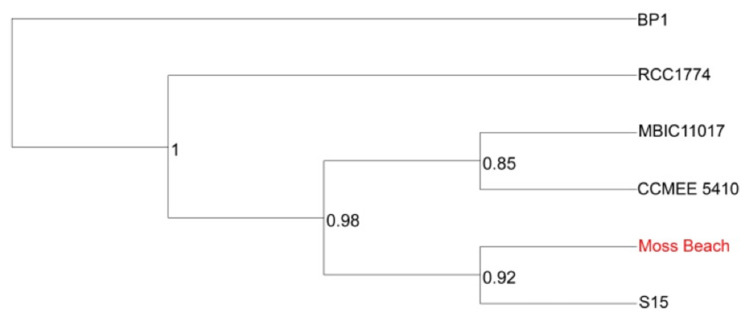
Phylogenetic tree of 6 concatenated OrthoFinder v2.5.4 [52] groups containing Species Tree inference from All Genes (STAG) support values at internal nodes. *Thermosynechococcus elongatus* BP-1 was used as an outgroup. The STAG support values represent the proportion of orthologous gene trees that were used to create the consensus tree that supports the particular branching pattern at that node.

Closely related strains Moss Beach and S15 shared approximately 5000 reciprocal best BLAST hits (Figure 10), as anticipated from their close AAI. Each genome codes for more than 1000 proteins that are not shared between the two strains, many of which were coded on plasmids. The comparison of orthologous proteins to other published *Acaryochloris* genomes is shown in Table S3 and the tree in Figure 11.

### 3.6. Light-Harvesting Genes

**Figure 12:**
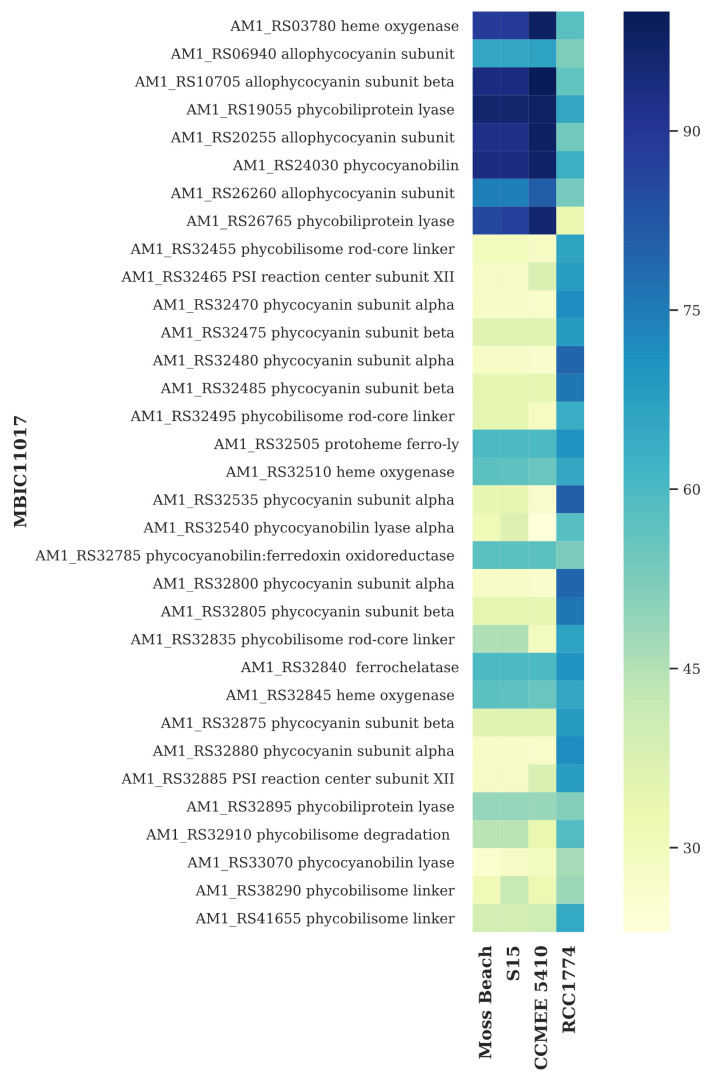
Heatmap displaying phycobiliprotein similarity of *Acaryochloris* species versus type strain MBIC11017 as the reference. Darker color indicates higher percent amino acid identity to the closest homolog in each of four strains (bottom).

Like the *Acaryochloris* type strain MBIC11017, the Moss Beach strain has two major light-harvesting and photoprotection systems along with accessory chlorophyll-binding proteins (CBPs) (Table S4). For the photosystem proteins and the CBP, a high percent identity suggests low divergence of these proteins between the two *Acaryochloris* strains. However, these two strains have a major difference in phycobiliprotein composition, as seen in the heat map in Figure 12. The divergence in phycobiliproteins in MBIC11017 is documented [24] and is a consistent feature in other Chl-*d*-containing *Acaryochoris* strains, though non-Chl *d*-producing strain RCC1774 does appear to have more similarity to MBIC11017 (Figure 12). Other than MBIC11017, all Chl-*d*-producing strains appear to produce only allophycocyanin, while, like MBIC11017, the non-Chl *d*-producing RCC1774 may also produce phycocyanin. As many of the large number of phycobiliproteins coded in MBIC11017 were found on plasmid pREB3 [25], the lack of plasmid similarity between strains likely accounts for this divergence in MBIC11017.

## 4. Discussion

**Algal host.***Acaryochloris* species or strains have been found as epiphytes on a variety of red algae: *A. marina* strain Awaji on the red alga *Ahnfeltiopsis flabelliformis* [15]; *Acaryochloris* sp. MPGRS1 on *Gelidium caulacantheum* [13]; *A. marina* S15 on *Pikea pinnata* [16,24], with three other *Acaryochloris* strains on red algae or red seaweed at the same site [24]. Ulrich, et al. [24] found at least ten more *Acaryochloris* strains on red algae from a wide sampling around Belize, Japan, the South China Sea, and the Arabian Sea. Manning and Strain [1] found Chl *d* on at least 18 species of red algae. From a preliminary diagnostic field trip we made to Moss Beach on 18 March 2015, we also found 16S rRNA evidence of *Acaryochloris* species on several algal hosts. A culture from an inoculation with a clipping of *Gelidium purpurascens* produced a 16S rRNA gene sequence with 99.65% sequence alignment with *Acaryochloris* sp. strain Awaji [15], a strain obtained from rinsing the surface of red macroalgae. A culture from inoculation with a clipping of *Cryptopleura ruprechtiana* yielded sequences with 100% coverage and 98% identity with *A. marina* CRS, *Acaryochloris* sp. CCMEE 5410, and *A. marina* MBIC 11017; 89% coverage and 99% identity with *Acaryochloris* sp. Awaji-1; and 99.6% identity with an unpublished sequence in the NCBI database (NIES 2412, Yamaguchi et al., submitted 6 October 2016). This 16S sequence also aligned perfectly with a culture from a clipping of *Polyneura latissima*.

From our August 2, 2015, field samples (Table 1), we used several algae species in common with those of Manning and Strain [1] as sources of inoculum, and additionally included brown algae. Far-red cyanobacterial cultures were successfully cultivated from both red and brown algae under the far-red light. As we only fully characterized one lineage of cultures from a brown alga, *Stephanocystis osmundacea,* we cannot assume that the Moss Beach site does not harbor multiple strains or species of far-red cyanobacteria, especially given that Ulrich, et al. [24] found four different *Acaryochloris* strains at the same Northern California site. The different modes of growth observed possibly suggest several different strains of *Acaryochloris* present or phenotypic plasticity within a strain. The broad occurrence of these far-red cyanobacteria across the several algae species also could indicate a cosmopolitan epiphyte growing on macroalgae in a variety of intertidal areas.

***In vivo* absorbance spectrum.** The *in vivo* Chl *d* Q_y_ absorbance peak of the Moss Beach culture occurred at 705 nm (obtained by gently vacuum-filtering whole cells onto GF/C filters) (Figure 5). This is much shorter in wavelength than the Q_y_ peak at 714–718 nm measured by both Miyashita, et al. [2] and Li, et al. [53] for the type strain of *Acaryochloris marina* MBIC11017 (obtained by measuring whole cells suspended in growth medium). Murakami, et al. [15] found their *Acaryochloris* strain Awaji (from red algae) displayed a Q_y_ peak at 711 nm (using a photodiode array detector under a microscope on whole cells grown under fluorescent light). A spectrum of whole intact cells of MBIC11017 measured on filter paper [9] displayed the Q_y_ peak at 710 nm. Mohr, et al. [14] measured an *in vivo* absorbance Qy peak at 707 nm in *Acaryochloris* sp. HICR111A, in a culture obtained from dead coral at Heron Island, Australia, and grown in near-infrared light centered at 720 nm. This shorter wavelength peak was attributed by Mohr, et al. [14] to the greater difficulty of maintaining homogenous cell suspensions while measuring the spectra of whole cells in glycerol (Wolfgang Hess, personal communication). However, Duxbury, et al. [54] used a Taylor sphere to measure the *in vivo* absorbance spectra of suspended whole cells (clumps loosened through brief bead beating or sonication; Min Chen, personal communication), observing the Q_y_ peak centered at 709 nm in MBIC11017 and at 707 nm in CCMEE 5410.

**Figure 13:**
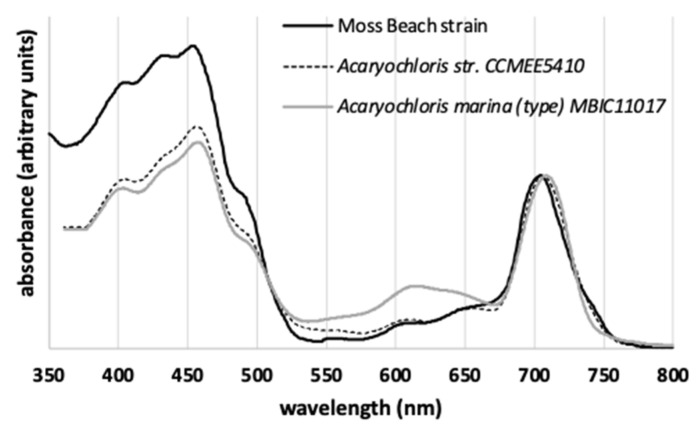
Comparison of the *in vivo* absorbance spectrum of the Moss Beach strain and those by Duxbury et al. [45] for *Acaryochloris marina* MBIC11017 and *Acaryochoris* str. CCMEE 5410. Only MBIC11017 shows the presence of phycobilins, manifested in the absorbance peak around 620 nm.

Duxbury, et al. [54] discussed the differences among studies that found the Q_y_ peak at longer wavelengths for MBIC11017, and noted that one such study [55] had an uncorrected baseline, while differences between strains could be due to small differences in Chl *d* pigment-binding proteins or to different contents of phycobilins. Thus, the differences observed in the Q_y_ peak wavelength for the same strain, as well as among the variety of strains of *Acaryochloris*, may be due to intrinsic differences between strains, culture conditions, pigment ratios (Chl *a*, Chl *d*, phycobilins), PSII/PSI ratios, differences between measurement techniques, as well as data smoothing. The Moss Beach strain’s *in vivo* absorbance spectrum is plotted together with that of MBIC11017 and CCMEE 5410 from Duxbury et al. [1] in (Figure 13). Thus far, it exhibits the shortest measured wavelength for the Q_y_ peak among known strains. The slight *in vivo* absorbance shoulder at 740 nm is not normally apparent in absorbance spectra of other *Acaryochloris* strains. It could be due to P740, but as yet we do not have an explanation.

**Chl *a*/Chl *d* ratio.** Previously reported Chl *a/d* ratios and Chl *d* percent of total chlorophylls reported in *Acaryochloris* include 0.03–0.09 or 92–97% Chl *d* [3], 0.016 ± 0.003 Chl *a*/Chl *d* or 84% Chl *d* [16], and 0.016–0.039 Chl *a*/Chl *d* or 96–98% Chl *d* [54] in *A. marina Str.* MBIC11017; 0.025 ± 0.0013 [16]; and 0.022–0.032 Chl *a*/Chl *d* or 97% Chl *d* in *A. marina Str.* CCMEE 5410. While other studies grew cultures in white fluorescent light, the study by Duxbury, et al. [54] had culture treatments in white fluorescent light, and LED light at 625 and 720 nm, with the maximum Chl *d* percent occurring with the far-red LED light. Mohr, et al. [14] compared the Chl *a/d* ratios of MBIC11017 and *Acaryochloris* sp. HICR111A under low, medium, and high light regimes in LED light at 720 ± 20 nm and found MBIC11017 was able to adjust the Chl *a/d* from ~1% to as high as ~5.7%. The HICR111A strain was less able to acclimate, with Chl *a/d* ranging 2–3%. They measured an *in vivo* absorbance peak of 707 nm for HICR111A vs. 710 nm for MBIC11017. Larkum, et al. [13] found up to 90% Chl *d* of all cellular chlorophyll in *Acaryochloris* sp. MPGRS1 from red algae in Australia [13] and an *in vivo* absorbance peak at 710 nm. The high Chl *d* content of the Moss Beach strain, with Chl *a/d* at 99%, was a little higher than but comparable to that of MBIC11017 grown under both white light [16] and far-red LED light [54]. Our LED environment compared to other studies was at the longest wavelength in the far red at 730 nm.

The shoulder that occurs in the HPLC chromatogram about 1 s after the Chl *d* peak does not occur in HPLC chromatographs of pigments extracts reported in Miyashita, et al. [3] or Larkum, et al. [13]. Further study would be needed to explain if it is Chl *d* or a degradation product. *A. marina* strain MBIC11017 also contained trace amounts of phycobiliproteins, a Chl *c*-like pigments, and α-carotene but not β-carotene [3].

**TEM.** TEM micrographs of the ultrastructure of different *Acaryochloris* species have been published for *A. marina* MBIC1017 [2,3,14,49,53,56,57,58,59,60], sp. CCMEE 5410 [16], sp. CCNUM4 [61], and sp. RCC1774 [26,49]. Direct comparisons of the imaged ultrastructural features, however, are complicated by different growth conditions (e.g., iron-rich, iron-poor, far-red vs. white light) and the methods of fixation. TEM images exist in the literature that used the same fixation methods as ours for MBIC11017 [3,26,49,57,60]. CCMEE5410 was post-fixed with permanganate by Swingley, et al. [26] and with osmium tetroxide (the same as our method) by Miller, et al. [16]. In our TEM images, the Moss Beach strain showed a parietal arrangement of the thylakoids, carboxysomes, and polyphosphate granules, features seen in other similarly prepared cells of *A. marina* strains MBIC11017 [2] and CCMEE 5410 [16]. MBIC11017 appears to have a tendency toward thylakoid membrane layers with wavy or “pinched” patterns (*sinsu* Swingley, et al. [26]), whereas CCMEE 5410 and the Moss Beach strain have the thylakoids parallel to the cells walls. However, we cannot conclude whether these are consistent differences or due to variation in growth conditions. CCMEE 5410 and the Moss Beach strain may be the most similar, possibly because MBIC11017 is the only strain to have phycocyanin. The Moss Beach strain does not show evidence of phycobilisomes, and we can also rule out their presence according to the absorbance spectra above and genome analysis below.

**Genome.** The high amino acid identity between strains Moss Beach and S15 suggests that both strains belong to the same species, identified as *Acaryochloris marina* in [24], which is in line with the shared 100% identity between Moss Beach and S15 16S rRNA genes and average nucleotide identity of 98%. While both strains were identified on the Pacific Coast of California, USA, the relatively high number of unique proteins in each strain (Figure 10), and lack of similarity in plasmid-encoded genes distinguishes these two as distinct strains. The S15 strain was isolated from Shelter Cove, Northern California, USA (40.022251° N, 124.0071373° W), while Moss Beach is located southward off the coast of Central California.

Phycobiliproteins aid in both light harvesting and photoprotection in most cyanobacteria. Phycobiliproteins were found to be associated with unique rod-shaped phycobilisomes in *A. marina* MBIC11017 from the Palau Islands, Japan [60]. *Acaryochloris* sp. MPGRS1 from red algae in a mangrove in Australia, was found to lack the gene for phycocyanin [13]. Miller, et al. [16] determined that *A. marina* strain CCMEE 5410 from the Salton Sea, California, does not produce phycobiliproteins. Mohr, et al. [14] found that *Acaryochloris* sp. HICR111A from Heron Island, Australia, lacks phycocyanin and phycoerythrin genes but has allophycocyanin genes. While type strain *A. marina* MBIC11017 produces a large amount of allophycocyanin and phycocyanin, as indicated by its dark color and signature “blue” phycobiliprotein absorbance peaks (see Figure 13 and Duxbury, et al. [54]), culture observation and spectra of strain Moss Beach suggest that this strain is significantly more deficient in these light-harvesting systems. Unlike MBIC11017, in which most of the phycobiliprotein-related genes are found on plasmid pREB3 [25], all of those in Moss Beach are coded on its main chromosome. Beyond the lack of phycocyanin-coding genes, Moss Beach only codes for a single set of genes for producing allophycocyanin, the most far-red light-absorbing phycobiliprotein, versus three copies of these genes in MBIC11017. This phycobiliprotein content was consistent with findings in other *Acaryochloris* strains [24], which suggested that phycocyanin-based light harvesting is exclusive to MBIC11017, allowing increased plasticity for acclimation to yellow light.

Given the high number of plasmids in the Moss Beach strain (and other *Acaryochloris* strains) compared to other bacteria, we checked whether any of these may be classified as chromids. Chromids are stable and essential extra-chromosomal replicons in bacteria [62], compared to the less stable plasmids, the latter being important agents for mediating lateral gene transfer [63,64]. We generated two dendrograms (not shown), by calculating distance based on tetranucleotide frequency between: (1) the chromosomes of MBIC11017, Moss Beach, and S15, and their respective plasmids; and (2) between the chromosome of Moss Beach and its plasmid. Both the dendrograms and a non-metric multidimensional scaling (NMDS) clustering analysis (not shown) showed no clear association between *Acaryochloris* chromosomes and any of the designated plasmids, suggesting they would not qualify as chromids. Harrison, et al. [62] found that about 10% of 897 fully sequenced bacteria possess chromids, among which were one genus of cyanobacteria, and that possession of chromids appears genus specific. Therefore, we conclude that the genus *Acaryochloris* does not have chromids.

## 5. Conclusions

We have isolated and identified an *Acaryochloris* from the site where chlorophyll *d* was originally discovered by Manning and Strain [1]. Whether this is the same organism from which these early researchers originally extracted Chl *d*, we cannot know, and it may be one of several Chl *d*-bearing strains or species coexisting at the site as epiphytes on multiple algae species.

Our strain is the first *Acaryochloris* to be isolated from a brown alga rather than red algae, although it is likely that it is also present on the red algae at the same site, from which far-red cyanobacteria cultures were also broadly obtained. Its *in vivo* spectral absorbance showed a Q_y_ peak centered at 704–705 nm, the shortest measured for *Acaryochloris* thus far. The range of values found in the literature both for the same strain as well as among strains has yet to be precisely explained. The Chl *d* content in the Moss Beach strain was very high, at 99% of total chlorophyll mass and a Chl *a*/Chl *d* ratio of 0.01, similar to that of MBIC11017. The TEM micrographs indicate the absence of phycobilisomes, which is further corroborated by genome analysis. The TEM imaging also suggests internal morphology closest to CCMEE 5410, as shown in Figure 2 from Miller, et al. [16].

Here, we contribute the full genome of the Moss Beach strain, which will add to the other three full genome sequences for *Acaryochloris*, the others being the type-strain MBIC11017, CCMEE 5410, and S15 [24,48]. The Moss Beach strain bears close 16S rRNA resemblance to the *Acaryochloris* sp. strain Awaji, discovered by Murakami, et al. [15] in Japan, for which there is no full genome sequence. However, it is closest in full genome comparison to strain S15 from the California coast [65], both epiphytes of red algae in the Pacific. Like strain S15, the Moss Beach strain also lacks complex phycobilisomes, as compared to type strain MBIC11017, perhaps indicating an adaptation to their similar red (or brown for strain Moss Beach) algal epiphyte spectral environment.

Given the multiple far-red cyanobacterial cultures obtained from red algae species at Moss Beach, in future work it remains to be seen whether they are the same strain as that isolated from the brown macroalga and characterized in this study, or whether there is differentiation by algae type. It also remains to be investigated whether the Q_y_ peak wavelength of the Moss Beach strain is sensitive to alternative growth conditions. There is an emerging wealth of *Acaryochloris* genome sequences that offer potential for additional comparative genomics, including data by Waterworth, et al. [51], other genomes linked to S15 [24], and an expected boom in more discoveries of far-red cyanobacteria. This new full genome sequence adds to the database for comparative studies to hopefully identify the elusive gene for Chl *d*.

Due to its close phylogenetic relationship to *Acaryochloris marina* strain S15, we designate this strain consistently as *Acaryochloris marina* strain Moss Beach. The genome is available under Genbank accession number GCA_021497025.1. Cultures may be obtained from the authors upon request.

## Figures and Tables

**Table 1:** Macroalgae species sampled from Montara State Marine Reserve, Moss Beach, California, USA on 2 August 2015. Column marked ‘c’ is for species from which enrichments produced far-red cyanobacterial cultures; ‘p’ for samples of algae clippings that were directly extracted and analyzed via HPLC. The species marked with an asterisk (*) are those for which cultures continue to be maintained. Further analyses in this paper were conducted on A-19, *Stephanocystis osmundacea*, a brown alga.

Sample No.	Species	Algae Color Class	c-Culture p-Pigment
A-1	*Plocamium pacificum*	red	
A-2	*Erythrophyllum delesserioides*	red	
A-3	*Microcladia coulteri*	red	
A-4	*Desmarestia herbacea*	brown	
A-5	*Costaria costata*	brown	
A-6	*Prionitis sternbergii*	red	C
A-7	*Farlowia compressa*	red	
A-8	*Cryptopleura lobulifera*	red	
A-9	*Neorhodomela larix*	red	
A-10	* Osmundea spectabilis	red	C
A-11	* Neogastroclonium subarticulatum	red	C
A-12	*Erythrophyllum delesseroides*	red	C
A-14	*Savoiea bipinnata*	red	C
A-15	*Sarcodiotheca gaudichaudii*	red	C
A-16	*Cryptopleura ruprechtiana*	red	C
A-17	*Chondracanthus canaliculatus*	red	C
A-18	* Chondracanthus canaliculatus	red	C
A-19	* Stephanocystis osmundacea	brown	p, c
A-20	*Ptilota densa*	red	C
A-21	*Cryptopleura ruprechtiana*	red	C
A-22	*Chondracanthus exasperatus*	red	
A-23	*Gelidium coulteri*	red	C
A-24	*Mazzaella flaccida*	red	
A-25	*Mazzaella splendens*	red	C
A-26	*Pikea californica*	red	C
A-27	*Mastocarpus jardinii*	red	C

**Table 2:** Genome features of strain Moss Beach (MB).

	Genome	pMB01	pMB02	pMB03	pMB04	pMB05	pMB06	pMB07	pMB08	pMB09	pMB10
Genome size	5,709,274	394,275	276,907	187,211	180,652	148,788	140,525	93,257	89,712	27,872	16,256
G+C content	47.01	45.67	46.16	46.61	46.32	45.52	45.52	44.53	44.34	42.33	42.14
Open reading frames	4589	311	212	155	143	128	114	73	81	25	12
Number conserved hypothetical	1075	143	63	75	81	71	77	41	53	17	5
Coding density	84.24	81.93	83.43	81.80	84.78	80.77	77.37	78.64	75.72	67.95	63.40
Average gene length	931	867	849	859	969	828	755	733	799	676	736
Ribosomal RNAs	6	0	0	0	0	0	0	0	0	0	0
Transfer RNAs	65	0	0	0	0	0	0	0	0	0	0
Pseudogenes	502	57	60	23	15	17	30	27	4	3	2
Insertion elements	150	40	39	9	6	12	12	23	3	1	1
Copy number (approximate)	1	1	1	1	1	1	1	1	1	1	1

**Table 3:** Amino acid identity (AAI) comparison of strain Moss Beach to other *Acaryochloris* strains.

Genome A	Genes in A	Genome B	Genes in B	Mean AAI (%)
Moss Beach	6583	S15	6307	97.6
Moss Beach	6583	CCMEE 5410	7493	89.1
Moss Beach	6583	MBIC11017	7495	88.9
Moss Beach	6583	SU_5_25	2261	76.4
Moss Beach	6583	CRU_2_0	4206	75.5
Moss Beach	6583	RU_4_1	4566	75.4
Moss Beach	6583	RCC1774	5531	65.8

## Data Availability

The genome is available under Genbank accession number GCA_021497025.1, submitted 18 January 2022 (https://www.ncbi.nlm.nih.gov).

## Supplementary Materials

The following supporting information can be downloaded at: https://www.mdpi.com/article/10.3390/microorganisms10040819/s1, Table S1: Genbank accession number; Table S2: GCpcnt; Table S3: Orthologous genes; Table S4: Phycobilins; Figure S1: ACT chromosome; Figure S2: ACT plasmids; Python scripts for genome analysis.
