# Supplementary material for: Discovery of Chlorophyll d: Isolation and Characterization of a Far-Red Cyanobacterium from the Original Site of Manning and Strain (1943) at Moss Beach, California
Source: Microorganisms. 2022 Apr 14;10(4):819. doi: 10.3390/microorganisms10040819 (PMC9029297; doi:10.3390/microorganisms10040819)
Supplement: Supplementary file 1 [file microorganisms-10-00819-s001.zip › microorganisms-1646849 -supplementary/Acaryochloris_MossBeach_Supplementary_Table-S1-S3_Figure-S1-S2_20220413.pdf]

microorganisms

MDPI

Supplementary Materials

Discovery of chlorophyll d: Isolation and characterization of a far-red cyanobacterium from the original site of Manning & Strain (1943) at Moss Beach, California

Nancy Y. Kiang [1,2,*], Wesley D. Swingley [2,3,*], Dikshyant Gautam [3], Jared T. Broddrick [4], Daniel J. Repeta [5], John F. Stolz [6], Robert E. Blankenship [7], Benjamin M. Wolf [7,†], Angela M. Detweiler [4,8,‡], Kathy Ann Miller [9], Jacob J. Schladweiler [3], Ron Lindeman [10] and Mary N. Parenteau [2,4,*]

1 NASA Goddard Institute for Space Studies, New York, NY, 10025, USA
2 Virtual Planetary Laboratory, Nexus for Exoplanet System Science, NASA Astrobiology Program, USA
3 Department of Biological Sciences, Northern Illinois University, DeKalb, IL, 60115, USA; gautam.dikshyant@gmail.com (D.G.); jjschlad1408@gmail.com (J.J.S.)
4 NASA Ames Research Center, Moffett Field, CA, 94035, USA; jared.t.broddrick@nasa.gov (J.T.B.); angeladetweiler@gmail.com (A.M.D.)
5 Woods Hole Oceanographic Institution, Woods Hole, MA, 02543, USA; drepeta@whoi.edu (D.J.R)
6 Department of Biological Sciences; Center for Environmental Research and Education, Duquesne University, Pittsburgh, PA, 15282, USA; stolz@duq.edu
7 Departments. of Biology and Chemistry, Washington University in St. Louis, St. Louis, MO, 63130-9899, USA;
reblankenship@gmail.com (R.E.B.); wolfbenjamin25@gmail.com (B.M.W.)
8 Bay Area Environmental Research Institute, Moffett Field, CA, 94035, USA
9 University Herbarium, University of California, Berkeley, CA, 94720, USA; kathyannmiller@berkeley.edu
10 Citizen Scientist, Alameda, CA, 94501, USA; ron.lindeman123@gmail.com
* Correspondence: nancy.y.kiang@nasa.gov (N.Y.K.); wswingley@niu.edu (W.D.S.); mary.n.parenteau@nasa.gov (M.N.P.)
† Current address: Pluton Biosciences, Inc., 11754 Westline Industrial Dr., St. Louis, MO 63146, USA.
‡ Current address: Chan Zuckerberg Biohub, San Francisco, CA, USA.

Citation: Kiang, N.Y.; Swingley, W.D.; Gautam, D.; Broddrick, J.T.; Repeta, D.J.; Stolz, J.F.; Blankenship, R.E.; Wolf, B.M.; Detweiler, A.M.; Miller, K.A.; et al. Discovery of Chlorophyll *d*: Isolation and Characterization of a Far-Red Cyanobacterium from the Original Site of Manning and Strain (1943) at Moss Beach, California. *Microorganisms* **2022**, *10*, 819. https://doi.org/10.3390/microorganisms10040819

Academic Editor: Firstname Lastname

Received: 5 March 2022
Accepted: 11 April 2022
Published: 14 April 2022

Publisher's Note: MDPI stays neutral with regard to jurisdictional claims in published maps and institutional affiliations.

Copyright: © 2022 by the authors. Licensee MDPI, Basel, Switzerland. This article is an open access article distributed under the terms and conditions of the Creative Commons Attribution (CC BY) license (https://creativecommons.org/licenses/by/4.0/)..

Supplementary Information

Table S1. Genbank accession numbers for strains used in this study

GenBank Accession number	Strains	Access date
GCA_000018105.1	*Acaryochloris marina* MBIC11017	15 May 2020
GCA_018336915.1	*Acaryochloris marina* S15	10 June 2021
GCA_021497025.1	*Acaryochloris* sp. 'Moss Beach'	18 January 2022*
GCA_003231495.1	*Acaryochloris* sp. RCC1774	15 May 2020
GCA_000238775.2	*Acaryochloris* sp. CCMEE 5410	15 May 2020
GCA_012032145.1	*Acaryochloris* sp. RU_4_1	15 May 2020
GCA_012034865.1	*Acaryochloris* sp. CRU_2_0	15 May 2020
GCA_012030725.1	*Acaryochloris* sp. SU_5_25	15 May 2020

*Submission date for *Acaryochloris* sp. 'Moss Beach'

Table S2. Guanine-cytosine (GC) percent content of strain Moss Beach compared to other *Acaryochloris* strains.

	**Moss Beach**	**MBIC11017**	**CCMEE 5410**	**S15**
Genome size (Mbp)	7.26	8.36	7.88	7.11
GC content (%)	45	47	47	45
ORFs	5843	8528	8383	6023
		new = 7046	new = 6948	

Table S3. Orthologous genes shared between *Acaryochloris sp.* Moss Beach against other published *Acaryochloris* strains.

**Genome A**	**Genes in A**	**Genome B**	**Genes in B**	**# orthologous genes**	**Orthologous fraction**
Moss Beach	6583	CCME 5410	7493	4214	64.01%
Moss Beach	6583	MBIC11017	7495	4206	63.89%
Moss Beach	6583	CRU_2_0	4206	2391	56.85%
Moss Beach	6583	RU_4_1	4566	2588	56.68%
Moss Beach	6583	SU_5_25	2261	1244	55.02%
Moss Beach	6583	RCC1774	5531	2813	50.86%

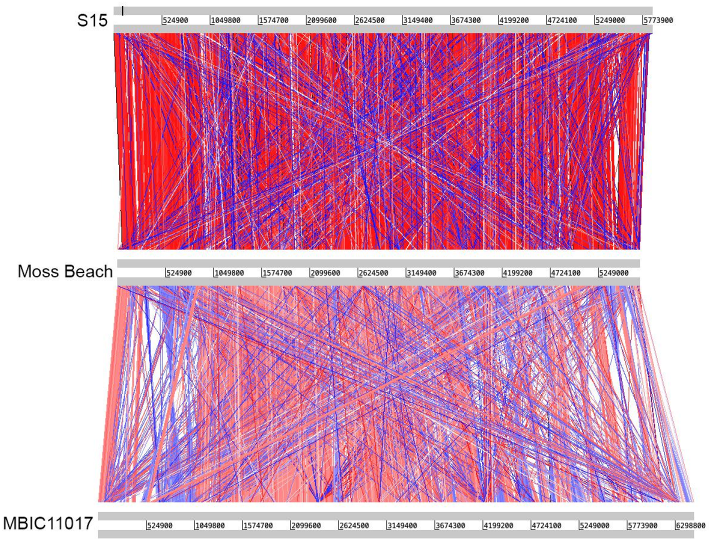

Figure S1. Artemis Comparison Tools (ACT) comparison between *Acaryochloris sp.* Moss Beach chromosome (middle) against *Acaryochloris sp*. S15 chromosome (Top) and *Acaryochloris marina* MBIC11017 chromosome (bottom) with a cut-off set to 70 percent identity. The red and blue bars show regions that are similar, with red bars indicating regions oriented in the same direction while blue bars indicating inverted orientation.

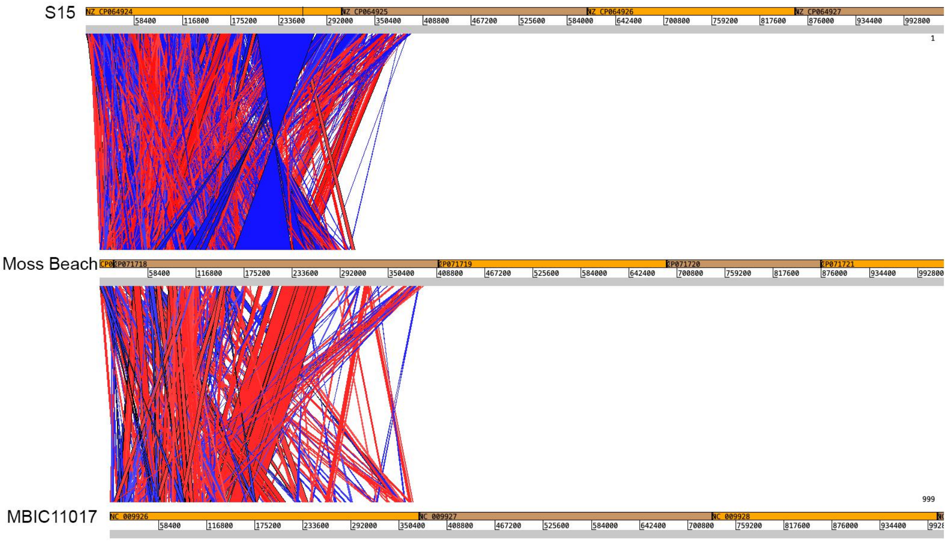

Figure S2. Artemis comparison tools (ACT) comparison between *Acaryochloris sp.* Moss Beach plasmids (middle), *Acaryochloris* sp. S15 plasmids (top), and *Acaryochloris marina* MBIC11017 plasmids (bottom) with a cut-off set to 70 percent identity. The red and blue bars show regions that are similar, with red bars indicating regions oriented in the same direction while blue bars indicating inverted orientation.
